# Childhood Adversities are Associated with Diabetes Management in Working Age in Finland

**DOI:** 10.1155/2014/864572

**Published:** 2014-04-28

**Authors:** Lauri Pisto, Atte Vadén, Lauri Sillanmäki, Kari Mattila

**Affiliations:** ^1^Department of General Practice, Medical School, University of Tampere, Lääkärinkatu 1, 33520 Tampere, Finland; ^2^Health Centre of Tampere, Finland; ^3^Hjelt Institute, University of Helsinki, Finland; ^4^Centre for General Practice, Pirkanmaa Hospital District, Tampere, Finland

## Abstract

*Backgrounds*. Research findings suggest that the mind can cause physical disease. To plan the best quality of care, general practitioner needs to understand an individual's health problems in physical, social, and psychological dimensions. This study sought to establish whether adverse life events occurring in childhood and adolescence are associated with diabetes. *Methods*. The cohort was collected from the health and social support (HeSSup) study—a postal follow-up survey of randomized working-aged Finns initiated in 1998. The response rate was 40.0% and the final cohort size 24057. Data on reimbursed diabetes medication during the years 1998–2006 were obtained from the Social Insurance Institute of Finland registers. Subjects were divided into insulin, tablet, combination therapy, and drug-naive groups together with a control group without diabetes. The prevalence of childhood adversities was assessed based on answers to six survey questions. *Results*. Childhood adversities showed predominant linkage to diabetes type 2 groups, especially to the combination therapy group requiring combined insulin and tablet treatment. No connection was found between childhood adversities and insulin use. Cumulative adversities did not markedly increase the association. *Conclusions*. Stressful events in childhood are associated with diabetes combination therapy in working age. The meaning of the relationship remains unsolved.

## 1. Introduction

Psychosocial factors may contribute to somatic illnesses through a variety of mechanisms. One recently revealed mechanism is a model based on epigenetic regulation and inheritance. The term epigenetics usually refers to changes in gene expression taking place without a change in the DNA sequence [1–3]. Today, the concept implies two main mechanisms: DNA methylation of cytosine bases and histone modification via acetylation and phosphorylation, which cause changes in gene activity. With chromosome duplication, such changes may be transferred to the ova or sperm. Childhood abuse, for example, has been shown to cause epigenetic changes in the expression of glucocorticoid receptors in the adult brain [4, 5]. As a result, the number of glucocorticoid receptors is reduced, this increasing the secretion of cortisol. This predisposes the individual to insulin resistance in adulthood.

Stress can be defined as any condition which seriously perturbs the physiological or psychological homeostasis of an organism [6]. Adversities ordinarily cause stress for a child [7, 8]. Stress caused by childhood events is usually by nature psychosocial. In such situations, the interpretation of what is experienced as stressful takes place in the brain [9]. Memory plays an essential role in psychosocial stress. During evolution, the ability to recall prior dangerous events has been a useful trait. We now know that traumatic childhood events affect the brain and thus an earlier experience may trigger a stress response later in life [5].

The significance of stress in the development of diseases has been described by, among others, the allostasis model. Allostasis refers to the homeostasis of stress or the regulation of stress in the body, enabling the individual to adapt to the stress factor [10]. When the body experiences long-lasting and too frequently recurring situations activating the stress response, a disease state may develop. This overload has long-lasting effects on the body's metabolic systems: endocrinological, immunological, and neurological. Studies have shown that these three systems are anatomically and functionally interlinked [11–13]. In consequence of this overload, the body is predisposed to a number of diseases common in the world today, one of them being diabetes type 2. For example, data from one longitudinal clinical study show that the prevalence of cardiovascular disease and diabetes is higher in later life among individuals separated from their families in early childhood during the Second World War [14].

Cortisol is an important hormone, which mediates the effects of stress in the endocrinological system. Hypothalamus, pituitary gland, and adrenal gland, that is, the HPA axis, regulate its secretion. Several brain areas, for example, the amygdala and hippocampus, which are activated during psychosocial stress, influence the hypothalamus [15]. Cortisol functions as an antagonist to insulin and alleviates the immune response [9, 16]. Epigenetic regulation of the receptor gene* NR3C1* leads to a decrease in glucocorticoid receptors, which in turn reduces the feedback from the HPA axis and thus increases cortisol secretion [4, 5]. This predisposes, among other things, to insulin resistance in adulthood.

When the immune system is activated, inflammation-mediating cytokines are secreted in the stress response. By inhibiting these cytokines, cortisol acts to restore the homeostasis of the organism after the stressful condition has subsided [10]. Without this effect, the inflammatory state would continue to destroy the organism [17]. Measured by CRP levels, as a result of earlier adverse childhood events the immune defence is more active in adulthood [18]. Chronic stress exposure may lead to lower cortisol levels, hypocortisolism, with increased Th1-mediated immune defence and an elevated risk of Th1-mediated autoimmune disease [19]. Cortisol reduces the function of the Th1-mediated immune defence [20]. Diabetes type 1 is mainly a Th1-mediated disease.

As the neurologic system is activated as the most immediate response to a stressful event, sympathetic activation induces the secretion of catecholamines in the cortex of the adrenal glands (the fight-or-flight response, that is, the acute stress response). Neurons also secrete neuropeptides and thus speed up recovery from the stress response and enhance adaptation to it [21, 22].

To plan the best quality of care, the general practitioner needs to understand an individual's health problems in physical, social, and psychological dimensions [23].

Today, nearly half a million Finns are estimated to have diabetes and the number is still steadily increasing [24]. There are only few high quality population studies on the state of diabetes care in Finland [25]. The management of the diabetes is emerging. During childhood, stress factors may influence the developing hormonal, neurological, and immunological systems and hence also their functioning in adulthood. Recent research findings suggest that stressful events can increase the risk of developing the difficult-to-manage form of diabetes, type 1 [26]. More researches on this aspect are needed.

In this population study, the aim was to establish whether there is a connection between stressful events during childhood and diabetes. The study hypothesis was that childhood adversities increase the prevalence of diabetes and come up in diabetes care.

## 2. Materials and Methods

### 2.1. Material

The health and social support study (HeSSup) was a prospective follow-up study of the psychosocial health of the Finnish working-aged population [27, 28]. The subjects belonged to a random sample drawn from the Finnish population register in four age groups: 20–24, 30–34, 40–44, and 50–54. The survey was carried out by postal questionnaire during 1998. Completed questionnaires were returned by 25 898 individuals, giving a response rate of 40.0%. The compatibility of the HeSSup sample with the Finnish general population was tested using official statistics, and the conclusion was that the differences in physical health between participants and the general population were small [27, 28]. A follow-up questionnaire (response rate 80.2%) was sent in 2003 to all who had responded in 1998.

The Social Insurance Institution of Finland (SII) maintains a nationwide database on all purchases of diabetes medication in Finland. For the purposes of this study, data on reimbursed diabetes medication in the period 1998–2006 were drawn from the registers of the SII and combined with the 1998 HeSSup data. The combined sample size was 24057, the number of drop-outs being 1841. These latter were individuals who did not grant us permission to combine their data with national registers or such as had dropped out because of death or had moved abroad. Medication purchases were encoded with ATC codes A10A (insulin) and A10B (oral diabetes medicine).

In the structured questionnaire, the participants were asked a diabetes-related survey question: “has a doctor ever told you that you have or have had diabetes?” Alternative replies were “yes” or “no”. Those who answered “yes” in 1998 or 2003 and those who had purchased diabetes medicine between the years 1998–2006 were classified as patients with diabetes. All in all, the cohort included 1182 patients with diabetes.

**Figure 1:**
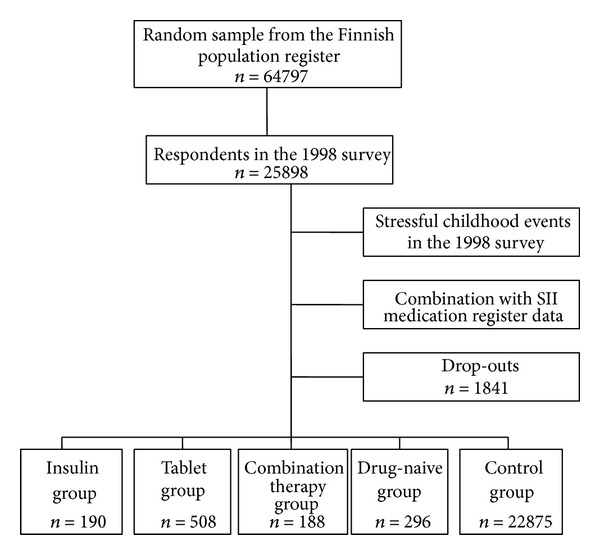
Flow chart illustrating the formation of study groups.

With the help of data from both the survey questionnaire and the SII registers, patients with diabetes were classified into five study groups (Figure 1), each patient with diabetes belonging to one group only. Those using only insulin medication (ATC code A10A) were classified into the insulin group (*n* = 190). It is likely that this group would be strongly weighted among those having diabetes type 1. People using oral diabetes medicine (ATC code A10B) were classified into the tablet group (*n* = 508) and consisted mostly of type 2 patients with diabetes. The third study group comprised those using both insulin (A10A) and tablet-form treatment (A10B) and was named as the combination therapy group (*n* = 188).

The fourth study group comprised those participants who in the 1998 survey reported having been diagnosed with diabetes by a doctor (*n* = 296). In a more detailed classification, we transferred a number of cases from this group into other groups based on their medication purchases. This group was termed the drug-naive group, and it included individuals who had reported themselves as patients with diabetes either a result of a misunderstanding with their doctor or whose condition was such that it could be treated with lifestyle and dietary changes without medication. It is also probable that this group included individuals who were not receiving any treatment at all and were managing their diabetes poorly as well as neglecting their medicine.

The control group (*n* = 22875) comprised individuals who were healthy with respect to diabetes and did not belong to any of the above-described four groups. In addition, they had no SII-registered diabetes-related entries nor subjectively reported diabetes in either of the surveys.

### 2.2. Methods

The subjects were asked to recall their childhood adversities in terms of the following structured questions: “did your parents divorce?”, “did your family have long-lasting financial difficulties?”, “did serious conflicts arise in your family?”, “were you often afraid of some member of your family?”, “was someone in the family seriously or chronically ill?”, “did someone in the family have problems with alcohol?” The alternatives were “yes”, “no”, or “I do not know”. Only the first two options were included in statistical analyses. The overall impact of these variables was estimated by the number of affirmative answers.

To confirm the reliability of responses regarding childhood adversities, the Cohen's kappa coefficient (*κ*) was used to assess associations between the 1998 and 2003 questionnaires. *κ* varied between 0.621 and 0.903 among participants.

The statistical significance of differences between the diabetes groups and the controls was tested by *χ* ^2^ test. Odds ratios (OR) with 95% confidence intervals (CI) were calculated by separate multivariate logistic regression analyses to measure the risk of belonging to a certain diabetes group. Associations between childhood adversities and diabetes study groups compared to controls were analysed with logistic regression methods as well. Each childhood adversity variable was analysed individually with each diabetes group as an outcome variable. Confounding factors, age, sex, Beck's depression inventory (BDI), body mass index (BMI), and alcohol usage, were changed into dichotomised variables for logistic regression and taken into account in the analyses (Table 1). The two youngest age groups (20–25 and 30–35) were merged into a group and the two oldest age groups (40–45 and 50–55) into another group. Weekly alcohol usage was likewise dichotomised into two groups: the “usage of 0–22 g” group and the “usage of over 22 g” group. The accumulation variable of childhood adversities was made by dichotomizing respondents into the 0-1 adversities and the 2–6 adversities groups. Analyses were made using the SAS system for Windows, release 9.2.

Respondents' demographic characteristics, age, sex, BMI, maximum BMI, BDI, smoking, and alcohol usage, are presented in Table 1 as frequency distributions.

The concurrent joint Ethics Committee of the University of Turku and Turku University Central Hospital considered approval not necessary for a normal cohort study, but all participants were requested to sign a consent form containing information on the study and to grant permission to allow subsequent studies with the same data set and the possibility to link up with national health registries.

## 3. Results

The prevalence of childhood adversities varied among the study subjects (Table 2). Compared to the control group, the drug-naive group reported most childhood adversities. Among the insulin-purchasing group (insulin group), only a family member's serious illness was more prevalent than among controls (9.7 percent units). The incidence of family members' serious illness was also statistically significant and 8.9–12.5 percent more prevalent in other study groups compared to the control population.

The insulin-purchasing group reported no long-lasting financial difficulties in childhood, whereas in other study groups financial difficulties were statistically significantly more prevalent than in the control group. Differences varied between 6.4–11.3 percent units. Among the groups using oral diabetes medicine, there had been fewer divorces than in the control population (5.1–6.7 percent units).

A family member's serious illness was the only childhood adversity which statistically significantly increased the risk of belonging to the insulin-managed diabetes group even when confounding factors were included (OR 1.89, 95% CI 1.17–3.04) (Table 3). Other childhood adversities and their accumulation were not connected with later-life insulin usage.

A family member's serious illness statistically significantly increased the risk of tablet-treated diabetes in adult life (OR 1.55, 95% CI 1.16–2.06). However, when confounding factors were taken into account in logistic regression analysis, the increase was no longer statistically significant (OR 1.24, 95% CI 0.92–1.67) (Table 3).

A family member's serious or chronic illness, long-lasting financial difficulties, and having been afraid of a family member statistically significantly increased the risk of having combination therapy, that is, a tablet- and insulin-treated form of diabetes later in life without controlling the confounding factors (OR 2.18, 95% CI 1.39–3.43, OR 2.29, 95% CI 1.45–3.62, and OR 1.92, 95% CI 1.08–3.40), respectively. When they were controlled, the risks were 1.70 (1.07–2.71), 1.90 (1.19–3.04), and 1.65 (0.91–3.00), respectively (Table 3). The connection remained statistically significant between childhood adversities and combination therapy diabetes, excluding the adversity of having been afraid of a family member. An accumulation of childhood adversities increased the risk of combination therapy diabetes (OR 1.86, 95% CI 1.03–3.38).

Having been afraid of a family member statistically significantly increased the risk of belonging to the drug-naive group (OR 1.64, 95% CI 1.10–2.45). However, when confounding factors in regression analysis were controlled, the connection was no longer statistically significant (Table 3).

## 4. Discussion

Childhood adversities are connected to diabetes, but in different ways depending on the etiology of the disorder. A family member's serious illness showed the most marked association with later life onset of diabetes, whereas an accumulation of childhood adversities was not markedly connected with the occurrence of diabetes.

A major strength of this study was that linkage to the SII data was successful for virtually all individuals in the original sample. Furthermore, the registry data on medicine purchases in Finland can be considered reliable and the data also verifies the diagnosis of diabetes. The large sample size ensured that conclusions drawn from the statistical analyses cannot be attributed to random effects. To obtain reliable information regarding an individual's exposure status, we analysed the kappa coefficient for every childhood adversity and, based on the results, our conclusion was that answers concerning childhood adversities can be considered reliable. Two analyses of respondents and nonrespondents of the HeSSup study were carried out to ensure better generalizability of the results [27, 28].

In previous diabetes research, childhood adversities have been connected with diabetes type 1 [19, 29, 30]. In this study, however, no clear connection between a childhood adversity and diabetes type 1 was found. The only clear risk factor for diabetes type 1 (the insulin group) seemed to be a family member's serious or chronic illness, which may point to the hereditary nature of the illness in the Finnish population.

On the other hand, childhood adversities would appear to be connected to the occurrence of diabetes type 2 (tablet group, combination therapy group, and drug-naive group). In previous research, the connection has been regarded as controversial [30].

Childhood adversities increased the risk of having a more advanced form of diabetes requiring combination therapy. A longer period of having diabetes, a poorly managed lifestyle, tablet-treated diabetes type 2, and metabolic syndrome in type 1 patients with diabetes are factors possibly leading to combination therapy treatment.

In the drug-naive group, all occurrences of childhood adversities except parental divorce were statistically significantly prevalent compared to controls. Drug-naive patients with diabetes had no entries of reimbursed diabetes medication purchases in the SII registers. There is thus no objective certainty that these participants do in fact have diabetes. Presumably in some members of this group the diabetes is managed by lifestyle and dietary changes only, while another part presumably neglects to take care of their diabetes and thus may not even have started the medication recommended by a doctor, or they are outside the health care system (i.e., unemployed and socially excluded). It is also possible that the drug-naive group includes individuals who recall negative life experiences better than other participants in the study. At least, according to the BDI questionnaire, compared to the other groups this group contained the greatest number of depressed participants, and, as is known, people when depressed tend to overemphasise negative life experiences in their past [31].

It has been shown that the effects of childhood adversities can be alleviated or even inhibited [9]. As they seem to increase the later life risk of developing the difficult-to-manage form of diabetes, it would be advisable to take childhood adversities into account in considering preventive health care, since the difficult-to-manage diabetes is expensive to treat for both society and the individual. For a general practitioner, to have a comprehensive approach to the care of an individual and a community, it is important to understand health problems in the individual's physical, psychological, social, cultural, and existential dimensions [23]. The emphasis should be, among the other support measures, on reducing the stress, that is, the allostatic overload caused by childhood adversities. In efforts to motivate those having the difficult-to-manage form of diabetes to avail themselves of treatment, health-care professionals might be better to concentrate on highlighting successes rather than adversities as people belonging to this group have already encountered an abundance of negative life experiences.

The aetiology of diabetes is partly unknown. It is also possible that the aetiology underlying the condition varies within and between diabetes groups [14]. Allostatic overload may complicate the progress of emerging diabetes by epigenetically activating or inhibiting genes. One possibility might be that the childhood adversities may be more markedly associated with challenging diabetes management than with the occurrence of diabetes. The combination therapy group may be weighted among those having more advanced or poorly managed diabetes [25]. According to recent research, childhood adversities are connected to poor self-care and diabetes, type 1, management as measured by haemoglobin A1 c concentrations [26]. As a conclusion, childhood adversities are associated with combined tablet and insulin-managed diabetes. The meaning of the relationship is not clear and is a subject for further research.

More research is warranted from the viewpoint of patients, from patients with problems and limitations. Successful care is the goal for both the family doctor and the patient with history and life experience. A competent family doctor keeps this in mind when taking care of diabetes patients.

## Figures and Tables

**Table 1:** Frequency distributions of the diabetes study groups and the control group in terms of respondents' demographic characteristics.

	Control group	Insulin group	Tablet group	Combination therapy group	Drug-naive group
	*n* = 22 875	*n* = 190	*n* = 508	*n* = 188	*n* = 296
	%	%	%	%	%
Gender					
Female	59.4	52.1	46.5	42.6	63.9
Male	40.6	47.9	53.5	57.4	36.1
Age in 1998					
20–25 and 30–35	51.9	59.5	15.5	13.8	32.4
40–45 and 50–55	48.1	40.5	84.5	86.2	67.6
BMI in 1998					
Under 25	62.9	56.6	16.4	9.7	36.0
25 or over	37.1	43.4	83.6	90.3	64.0
BMI maximum in 1998					
Under 25	46.7	35.8	9.6	4.4	22.5
25 or over	53.3	64.2	90.4	95.6	77.5
BDI in 1998					
Under 19	95.7	93.0	90.0	88.7	87.8
19 or over	4.3	7.0	10.0	11.3	12.2
Smoking in 1998					
Does not smoke	45.4	38.7	37.1	30.4	34.2
Smokes or has quit	54.6	61.3	62.9	69.6	65.8
Weekly drinking					
0–22 g	30.4	33.2	32.9	37.4	37.2
Over 22 g	69.6	66.8	67.1	62.6	62.8

**Table 2:** Occurrence of childhood adversities (%) in the diabetes study groups and the statistical significance of differences from controls.

	Control group	Insulin group		Tablet group		Combination therapy group		Drug-naive group	
	*n* = 22 875	*n* = 190		*n* = 508		*n* = 188		*n* = 296	
	%	%	*P* value	%	*P* value	%	*P* value	%	*P* value
Parental divorce	17.4	16.8	0.835	**12.3**	**0.004**	**10.7**	**0.019**	18.0	0.782
Long-lasting financial difficulties	24.4	23.7	0.821	**30.8**	**0.001**	**35.7**	**<0.001**	**33.1**	**0.001**
Serious family conflicts	24.8	20.1	0.141	27.7	0.141	25.9	0.723	**31.7**	**<0.001**
Afraid of a family member	12.8	10.9	0.459	14.3	0.312	16.3	0.152	**22.2**	**<0.001**
Seriously or chronically ill family member	24.9	**34.6**	**0.002**	**33.8**	**<0.001**	**37.4**	**<0.001**	**36.0**	**<0.001**
Alcohol problem of a family member	24.0	27.3	0.293	26.5	0.192	24.7	0.817	**31.8**	**0.008**

**Table 3:** Summary of separate multivariate logistic regression analyses with odds ratios and 95% confidence intervals (OR; 95% CI). Confounding factors; age, sex, Beck's depression inventory (BDI), body mass index (BMI), and alcohol usage were included in the analyses.

	Insulin group	Tablet group	Combination therapy group	Drug-naive group
	OR (95% CI)	OR (95% CI)	OR (95% CI)	OR (95% CI)
Parental divorce	0.85 (0.48–1.50)	1.00 (0.69–1.46)	1.16 (0.64–2.08)	1.31 (0.90–1.91)
Long-lasting financial difficulties	0.84 (0.53–1.34)	1.04 (0.78–1.40)	**1.90 (1.19**–**3.04)**	0.96 (0.69–1.33)
Serious family conflicts	0.77 (0.45–1.33)	1.24 (0.88–1.75)	0.65 (0.36–1.18)	0.86 (0.58–1.28)
Afraid of a family member	0.85 (0.44–1.67)	0.99 (0.67–1.45)	1.65 (0.91–3.00)	1.43 (0.95–2.16)
Seriously or chronically ill family member	**1.89 (1.17**–**3.04)**	1.24 (0.92–1.67)	**1.70 (1.07**–**2.71)**	1.09 (0.78–1.53)
Alcohol problem of a family member	1.29 (0.79–2.11)	1.12 (0.82–1.53)	1.25 (0.75–2.06)	1.03 (0.72–1.46)
2–6 childhood adversities	0.89 (0.51–1.57)	1.36 (0.95–1.95)	**1.86 (1.03**–**3.38)**	0.70 (0.46–1.07)
